# Supplementary material for: Diabetes management cascade in Ulaanbaatar, Mongolia
Source: PLoS One. 2025 Dec 30;20(12):e0337413. doi: 10.1371/journal.pone.0337413 (PMC12752991; doi:10.1371/journal.pone.0337413)
Supplement: Attachment — Submitted filename: Rebuttal letter .pdf [file pone.0337413.s002.pdf]

Onom Fouandation, National Academy street, 18th Khoroo,
Sukhbaatar district, Ulaanbaatar 17011, Mongolia
Phone: +976 70122006

E. team@onomfoundation.org W. www.onomfoundation.org

REBUTTAL LETTER

Date 07/05/2025

Academic Editor

PLOS One

Re: Manuscript ID /PONE-D-24-39264/

Diabetes management cascade in Ulaanbaatar, Mongolia

Dear Editors,

Thank you for the opportunity to revise our manuscript and for the thoughtful and constructive comments provided by the reviewers. We appreciate the time and effort invested in evaluating our work. We have carefully considered all the comments and revised the manuscript accordingly. Below, we provide a detailed, point-by-point response to each comment.

For clarity, we have included the reviewer/editor comments in **bold**, followed by our responses in *plain text*. All changes made in the manuscript have been highlighted in the revised version with tracked changes.

Reviewer 1 Comments: Line 58

While IFG is a criterion for diagnosing, it's not synonymous with diabetes itself (which can also be diagnosed via HbA1c as well). It would be better to include the exact definition and cut-off points of diabetes from STEPS.

Response: We appreciate the reviewer's important observation. In response, we have clarified the diagnostic criteria used in the STEPS survey, including the specific cut-off points for impaired fasting glucose (IFG) and diabetes. We have also acknowledged that IFG is one of several diagnostic criteria and not equivalent to a full diabetes diagnosis, which may also include HbA1c-based criteria.

Comments: Line 62

the KAP survey data is from 2010. While it highlights a historical lack of awareness, it is dated. If more recent studies are available, please include and cite them as well. If not, briefly acknowledge the age of this study while stating it is the best available indicator of past awareness levels.

Response: We thank the reviewer for this thoughtful comment. We have searched for more recent national KAP surveys related to diabetes in Mongolia; however, to our knowledge, no comprehensive studies have been conducted since 2010. We have therefore acknowledged the age of the 2010 KAP data in the revised manuscript and clarified that, while dated, it remains the best available indicator of public awareness at that time.

Comments: Line 96

"For the laboratory analysis, one in two of the selected participants aged were recruited" Please specify the exact age range subjected to this selection for lab tests and provide some explanation about how the one in two were selected. Was it random or based on other criteria?

Response: Thank you for the helpful suggestion. We have clarified the exact age range of participants selected for laboratory testing and provided details on the sampling method. Specifically, participants aged 20 and above years were eligible for the STEPS survey, and among them, every second participant (1 in 2) was selected for laboratory testing using a systematic sampling approach.

Comments: Line 151

Specify if any standard population structure were used to standardize the prevalence (WHO standard population etc.)

Response: We appreciate the reviewer's comment. We have revised the manuscript to specify that the age-standardized prevalence estimates were calculated using the World Health Organization (WHO) world standard population as the reference.

Comments: Table 3

"parity" section seems incorrect considering the subheadings area marital status.

Response: Thank you for pointing this out. We have corrected the heading to accurately reflect the content as "Marital Status."

Comments: Line 400

fix "the with the overall minimal presence"

Response: Thank you for pointing this out. We have corrected the grammatical issue to improve clarity and readability.

Comments: Line 407

Could you briefly explain in the manuscript on why these supply-side gaps might exist in the Mongolian context. Is it lack of specific national guidelines for diabetes management in primary care? Limited resources for training primary care physicians? Issues with laboratory access/standardization for monitoring (relevant given the HbA1c limitation discussion)? Lack of integrated health information systems?

Response: Thank you for this insightful comment. We have expanded the discussion in the manuscript to provide context on the potential causes of the supply-side gaps in diabetes management in Mongolia. Specifically, we acknowledge the challenges related

to the lack of national guidelines for diabetes management, limited resources for training primary care physicians, laboratory access issues, and the lack of integrated health information systems, all of which could contribute to the observed gaps.

Reviewer 2 Comments:

Good paper with enough information. However there are some points needed to modify. Introduction: the paragraphs are not related to each other. Please make them in better order with relation sentences at the end of the each paragraph. Method: it is not clear what do you want to do. Please explain more about the method section. Result: I think this section is not in good order. Please clarify the outcome measures and explain the results in appropriate way. Discussion: I did not see the strength and limitation of your study.

Response: We sincerely thank the reviewer for the thoughtful feedback. We have revised the Introduction to improve the logical flow between paragraphs. Transitional sentences were added at the end of each paragraph to ensure that the sections are well-connected, making the argument clearer and more cohesive.

We have expanded the Methods section to clarify the study's objectives, the participant selection process, and the laboratory testing procedures. Additionally, we have provided more detail on how the sampling was conducted, including the inclusion and exclusion criteria.

We appreciate the reviewer pointing this out. We have added a new subsection titled Strengths and Limitations *at the end of the Discussion. This section highlights the key strengths of our study, such as its large sample size and national scope, as well as limitations, including the use of self-reported data and the lack of access to HbA1c testing.*

Sincerely Yours,
Naranjargal Dashdorj, Chief Executive Officer
Phone: +976 99199246
Email: dashdorj@onomfoundation.org
