# Supplementary material for: Diabetes management cascade in Ulaanbaatar, Mongolia
Source: PLoS One. 2025 Dec 30;20(12):e0337413. doi: 10.1371/journal.pone.0337413 (PMC12752991; doi:10.1371/journal.pone.0337413)
Supplement: Attachment — Submitted filename: Rebuttal_letter__auresp_2.pdf [file pone.0337413.s003.pdf]

ONOM FOUNDATION

Onom Fouandation, National Academy street, 18th Khoroo,
Sukhbaatar district, Ulaanbaatar 17011, Mongolia
Phone: +976 70122006

E. team@onomfoundation.org W. www.onomfoundation.org

REBUTTAL LETTER

Date 28/09/2025

Academic Editor

PLOS One

Re: Manuscript ID /PONE-D-24-39264/

Diabetes management cascade in Ulaanbaatar, Mongolia

Dear Editors,

Thank you for the opportunity to revise our manuscript and for the thoughtful and constructive comments provided by the reviewers. We appreciate the time and effort invested in evaluating our work. We have carefully considered all the comments and revised the manuscript accordingly. Below, we provide a detailed, point-by-point response to each comment.

For clarity, we have included the reviewer/editor comments in **bold**, followed by our responses in *plain text*. All changes made in the manuscript have been highlighted in the revised version with tracked changes.

Reviewer Comments:

Comment: Add a brief note comparing your findings to similar published work (if available) to strengthen context.

Response:

We have expanded the Discussion section to include a comparison of our results with recent studies on diabetes prevalence and risk factors in similar populations. This provides greater context and highlights consistencies and differences with previous research.

Comment: Include more explicit acknowledgment of study limitations (e.g., sample size, potential selection bias).

Response:

We have added a dedicated paragraph in the Discussion section explicitly outlining the study limitations, including the moderate sample size and potential selection bias due to recruitment methods. These acknowledgments emphasize the need for cautious interpretation and suggest directions for future research.

Comment: “Ensure all figure legends are self-explanatory.”

Response:
We have revised all figure legends to be fully self-explanatory. Each legend now clearly describes the content of the figure, including sample sizes, variables measured, units, and any abbreviations used, so that the figures can be understood independently of the main text.

Comment: Abbreviations should be defined at first use within tables and figures.
Response:

We have defined all abbreviations at their first appearance within tables and figures, either in the legend or footnotes, for clarity and reader convenience.

Comment: In the abstract and results, avoid redundancy (e.g., “increase in levels was increased”).
Response:

We carefully reviewed the manuscript and rephrased redundant expressions to improve clarity and conciseness, including in the Abstract and Results sections.

Sincerely Yours,
Naranjargal Dashdorj, Chief Executive Officer
Phone: +976 99199246
Email: dashdorj@onomfoundation.org
